# Exposed Pulps, and How Nature Cures Them

**Published:** 1877-02

**Authors:** M. C. Sim


### EXPOSED PULPS, AND HOW NATURE CURES THEM.

BY M. C. SIM, D. D. S.

A dentist does not need to apologize for writing on a subject connected with his every day life and experience, nor for writing on a subject so intimately connected with his usefulness and success as that of pulp exposure and its treatment.

Mr, S. S. White recognized the importance of the subject when he propounded the following queries, which have been answered by all thinking dentists according to their own knowledge and experience, but differing very materially as to results, modes of treatment, and physiological conditions.

"The pulp of a tooth being deprived of a portion of its normal protecting wall, it has been observed that tissue analogous apparently to dentine, has been deposited in the orifice, closing it entirely, and thus restoring the condition necessary to the preservation of the pulp.

"This result having occurred occasionally, it is important to know whether any one substance used as a covering, has been more frequently followed by such deposits than when others have been used.

"Is the necessary amount of lime held in solution in the blood of all healthy persons? If so, how can the vital forces of the part be stimulated to make a reparative deposition, as is done when fractured bones are properly set and protected?

"Can this action be induced, or is it only necessary in order to obtain such deposition generally, that pulps should be freed from all irritating agents and be protected by a wall securing it from foreign substances and abnormal thermal changes?"

These are the queries and in answering them I only give the result of my knowledge and experience.

To the first question I answer, Yes. The system in health contains all the elements necessary to the support of all the tissues, so that although the decay of teeth may indicate a lack of the necessary elements during the formation of them, yet the person does not exist who having the usual and ordinary functions of digestion and assimilation, and who can get proper food, has not the elements necessary for the protection of an exposed pulp by reparative deposition.

How can the vital forces of the part be stimulated to make a reparative deposition, as is done when fractured bones are properly set and protected?

The exposure of the pulp is of itself sufficient stimulation to the vital forces of the part, to induce reparative deposition when properly protected, as fracture of the bone is; the inflammation following it being a promoter of reparative deposition "if properly set and protected."

It can not be said that "any one substance has been more frequently followed by such deposits than when others have been used," because that anything having certain known physical properties, which adapt it to that purpose, may be used successfully, so that if any one thing has been more successful than another, it is that which has been used more than any other.

This reparative deposition can not be induced, if under favorable outside conditions there is no deposition, then the treatment must be constitutional, and in most cases a failure. Substances recommended for inducing reparative deposition, are just as efficient, and no more so, than a simple inert substance used merely as a protection or covering.

It is "only necessary to free it from all irritating agents, (the most irritating being decomposed dentine) protect it by a wall securing it from foreign substances and abnormal thermal changes," leaving the "reparative deposition" in the hands of Nature who doeth all things well.

The cap or covering then becomes of prime importance, as it must be hard enough to protect from injury, must not readily conduct heat or cold, must be by nature or otherwise adapted to that use, and contain nothing injurious to the delicate, sensitive, little organ we call the pulp.

What then best fulfills all these requirements?

Twenty years ago Dr. Watt covered or protected pulps with a solution of gutta percha, and this practice has been followed ever since with more or less success. In fact it was with this I made my first efforts, but I found that of pulps, apparently in the same condition, one would bear it while another would not.

The substance next used was the oxide of zinc, with carbolic acid, this was successful so far as the contact with the pulp was concerned, but it was difficult to keep in place while filling over it, as I frequently did, especially in the anterior teeth.

Finally Fletcher's Artificial Dentine was recommended to me, and after a thorough trial I use it almost to the exclusion of everything else. Not knowing its composition, and not wishing to use anything of which I was ignorant, I corresponded with Mr. Fletcher, who I found ready to give the desired information. He says, under date of July 22; "In reply to your note the artificial dentine is a basic sulphate of zinc, the acid being partially combined with the oxide when sent out, or rather the sulphuric acid is neutralized with oxide of zinc until it admits of being dried. and the remainder of the oxide is then added. It combines and hardens almost instantly with water, and I use a solution of gum arabic to stop setting until you have time to work it."

Thus you see there is no medicinal effect, none is intended; it is simply an inert mass placed in contact with the healthy but abnormal, because exposed pulp, intended simply to protect it until nature furnishes a better protector. Among them these may be mentioned as advantages it has over others. It will adhere to dry dentine; it becomes sufficiently hard in a few moments to allow the building of gold over it without danger of crushing; it is practically a non-conductor of heat and cold, (I judge of this by the effect and not by a practical experiment) and any exposed pulp in a healthy condition will bear it in contact with it.

It is not a difficult thing to do, nor does it require so much skill to cap or protect a healthy exposed pulp for a healthy person, as to determine the exact pathological condition, in which a person or pulp may be, and indicate the treatment, constitutional and local, necessary to restore both to a normal condition, which is absolutely necessary to success in treatment of diseased teeth or exposed pulps.

As for the "reparative depositon" over the point of exposure I say this. Formerly I filled all teeth in which pulps were capped with a temporary filling and examined them at times, so that I have seen it from the soft sensitive bluish tinged dentine, to that of intense hardness, not sensitive under the excavator, and of the same color as the dentine of the tooth.

For some time I have not filled temporarily, except those in which from some known idiosyncrasy, there was doubt of the success of the operation, or an unusually large exposure making it hazardous to build over it.

Of course some die. The treatment now recommended for diseased pulps having proved a failure in my hands, never having saved an inflammed pulp, chronic inflammation I mean, those in which there was a continuous pulsating pain, only easy occasionally and then but for a few moments, the exposure of long standing hypersensitive dentine around it, and a colorless fluid oozing from it. Pulps in that condition I have never with any kind of treatment succeeded in keeping alive, apparently death commences or life ceases at the point of exposure extending into the root or roots from that.

I have not at present the exact figures to calculate the proportion of successful cases to those operated on, but from a arge number now in my mind I can say it is well worth any person's best efforts, but don't get discouraged if you fail, you will fail sometimes and when you do, strive to learn why, and the next effort will be worth more because you have, by knowing why you failed, lessened the risk of failing again.

There are in this jumble of facts and assertions, of success and failure, of longing and effort, many things that need the critic's eye, but there is nothing new or startling, and so the dentist who feels, as only a dentist can feel, that the success, elevation and scientific education of the future dentist is foreshadowed in the young men of the present, will have only kind criticisms, gentle admonitions and well wishes.

But as this is to the young men of the professiom, from one of them let not any of the older men withhold their exposition of error, and by so doing seem to acquiesce in what may lead to grave and irretrievable harm.

